# Novel multifunctional RKKY coupling layer for ultrathin perpendicular synthetic antiferromagnet

**DOI:** 10.1038/s41598-018-29913-6

**Published:** 2018-08-06

**Authors:** Jyotirmoy Chatterjee, Stephane Auffret, Ricardo Sousa, Paulo Coelho, Ioan-Lucian Prejbeanu, Bernard Dieny

**Affiliations:** Univ. Grenoble Alpes, CEA, CNRS, Grenoble-INP, INAC-SPINTEC, Grenoble, France

## Abstract

A novel multi-functional antiferromagnetic coupling layer (MF-AFC) combining Ru and W is revealed to realize an extremely thin (3.8 nm), back-end-of-line compatible as well as magnetically and electrically stable perpendicular synthetic antiferromagnetic layer (pSAF), essential for spintronic memory and logic device applications. In addition to achieving antiferromagnetic RKKY coupling, this MF-AFC also acts as a Boron sink and texture-breaking layer. A detailed optimization of the thickness of the various involved layers has been carried out to obtain extremely thin-pSAF reference layer with stable magnetic properties, which enables the realization of sub-20 nm STT-MRAM cells. Two important advantages are provided by this ultrathin reference layer: the easing of the reference layer etching and the minimization of the dipolar field acting on the storage layer magnetization.

## Introduction

Perpendicular magnetic tunnel junction (pMTJ) stacks are the storage elements of STT-MRAM. Among the various technologies of non-volatile memories, STT-MRAM gathers a unique combination of assets: non-volatility, write speed (3–30 ns), density, low consumption (a few tens of fJ/write), and very importantly an extremely long write endurance (>10^13^cycles)^1–5^. In order to fabricate a high density and high capacity memory array at advanced technology node (sub-20nm cell diameters), the stack must be as thin as possible, especially below the tunnel barrier. Indeed, for patterning the magnetic tunnel junction stacks, physical etching by ion beam (IBE) remains the preferred approach. However, this technique does not allow to reach very narrow pitch due to redeposition of non-volatile etch products at the side walls of the memory cells and shadowing effect when etching at large incidence angle^6,7^. Reducing the thickness of the bottom part of the stack below the tunnel barrier is therefore very advantageous to reduce the amount of redeposited species on the sidewalls of the MTJ. This increases the yield, the magnetoresistance amplitude, and decreases the dot-to-dot variability^8^. Besides, the MTJ stack must endure a back end of line annealing at 400 °C, exhibit a thermal stability factor between 60 and 100 depending on the memory capacity and acceptable error rate over the operating range of temperature, have low Gilbert damping in the storage layer, a high spin transfer efficiency to minimize write current and a large tunnel magnetoresistance amplitude (TMR > 200%) to maximize read speed^8–11^.

Conventional pMTJ stacks comprise a relatively thick pSAF of composition: *buffer layer*/[*Co*/*X*]_*m*_/*Co*/*Ru/*[*Co/X*]_*n*_/*Co*/*texture breaking layer*/*FeCoB*/*tunnel barrier*, where X represents Pt, Pd or Ni metals. Thinner pSAF structures composed of *buffer layer*/[*Co/X*]*m/Co*/*Ru*/*Co*/*texture breaking layer*/*FeCoB*/*tunnel barrier* were proposed to contribute reducing the generation of non-volatile etch product during etching^12^. However, these thin pSAF stacks do not exhibit sharp magnetic reversal with high squareness after annealing at 400 °C temperature^13^. This is most likely due to inter-diffusion of the texture breaking material (for instance Ta) into the FeCoB layers.

In this paper, we report on an innovative way to achieve extremely thin, magnetically stable and thermally robust pSAF using a novel multi-functional anti-ferromagnetic coupling layer (MF-AFC) based on Ru/W bilayers. The resulting pSAF has a composition of the form *Hard layer* (*typically a* (*Co/Pt*) *based multilayer*)*/MF-AFC/FeCoB/MgO*. In contrast to the well-known RKKY coupling layer, such as Ru, Ir etc.^14,15^, these novel hybrid MF-AFC are realized by combining two different materials from Ru, Ir, Rh etc. and W, Mo, Nb, Ta etc. to achieve multi-functional properties. Apart from achieving antiferromagnetic RKKY coupling, the other functionalities are (i) to absorb boron out of the magnetic electrode (FeCoB) in contact with the MgO barrier upon annealing of the stack, (ii) to allow the crystalline transition between the fcc part of the stack of 3-fold symmetry and the bcc part of the stack next to the MgO barrier (of 4-fold symmetry) and (iii) to prevent interdiffusion between the two parts of the SAF during high temperature annealing. This coupling layer suppresses the need for an extra magnetic layer and texture breaking layer between the RKKY coupling layer and the FeCoB reference layer. As a result, the thickness of the pSAF layer can be significantly reduced resulting in easier etching and lower dipolar field exerted by the pSAF layer on the storage layer.

Along the paper, the reference layer is also called polarizer layer.

## Results and Discussions

### Dipolar field in macrospin approximation

**Figure 1:**
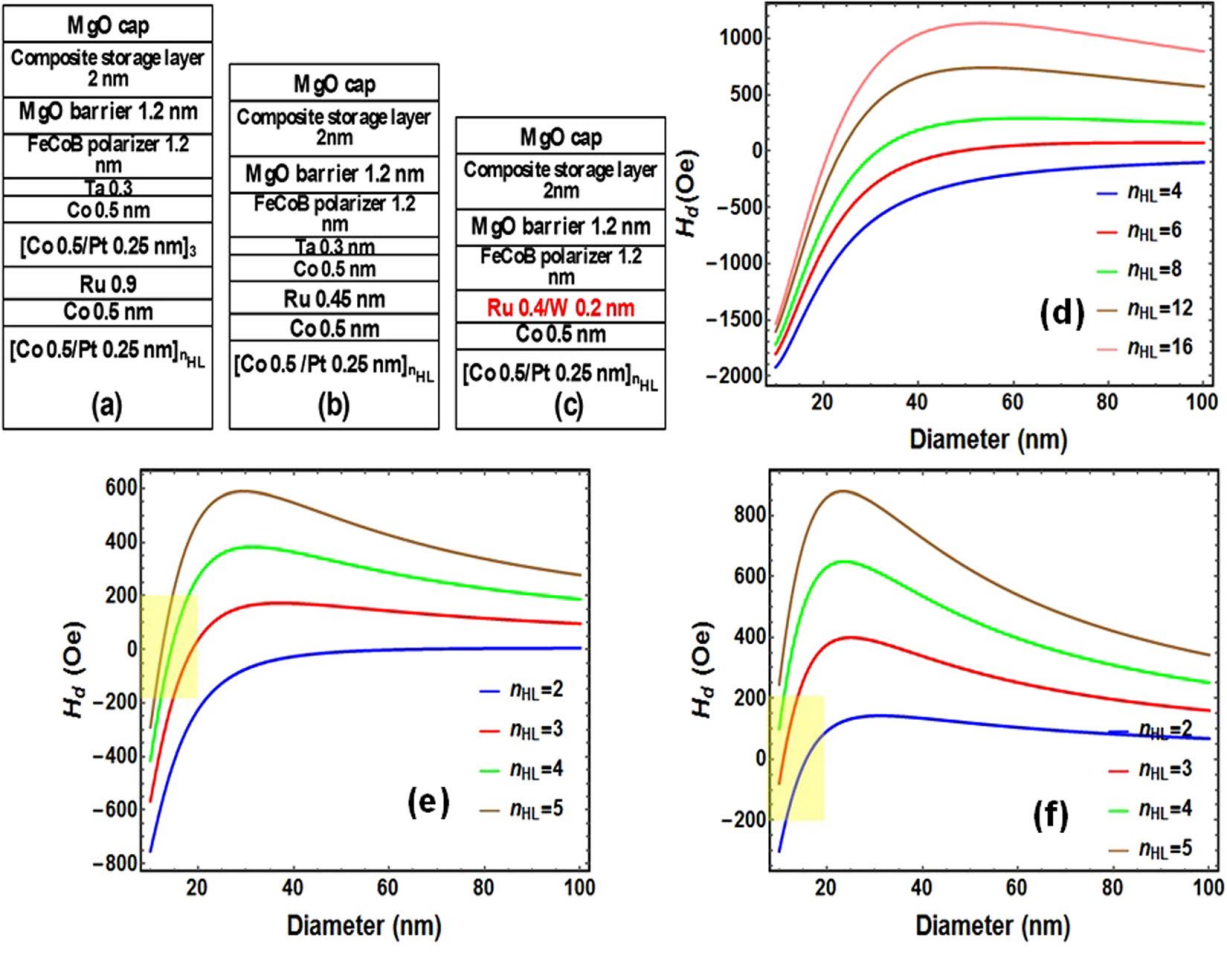
Configuration of (**a**) A conventional thick pSAF using two sets of Co/Pt MLs, antiferromagnetically coupled by the Ru RKKY coupling layer. (**b**) A previously proposed thin-pSAF with Co/Pt MLs as a hard layer, antiferromagnetically coupled with Co/Texture breaking layer (Ta)/FeCoB polarizer layer by Ru. (**c**) A thin pSAF with multi-functional RKKY coupling layer (Ru/W). (**d**–**f**) Dipolar field (H_d_) at the center of storage layer from the pSAF as function of device diameter and for different numbers of Co/Pt bilayers in the hard layer (n_HL_), calculated in macrospin approximation for the above mentioned three types of pSAF layers respectively.

Three types of pMTJ stacks are shown in Fig. 1: pMTJ with (a) conventional thick pSAF, (b) previously proposed thin-pSAF with separated RKKY coupling layer and texture breaking layer^12^ and (c) novel thin-pSAF with multi-functional RKKY coupling layer (MF-AFC). The dipolar field (*H*_*d*_) from the pSAF layer at the center of the storage layer (SL) was calculated in macrospin approximation as a function of cell diameter. The results are shown in Fig. 1(d–f). It is obvious from Fig. 1(d), that maintaining low stray field (<200 Oe) for sub-20 nm memory cell is impossible with such conventional thick pSAF layer even by increasing the number of Co/Pt bilayers (*n*_*HL*_) in the bottom part of the SAF up to 16 or even more. Figure 1(e,f) show *H*_*d*_ versus device diameter respectively for the previously proposed thin-pSAF and for the novel thin-pSAF with MF-AFC, which are schematically depicted in Fig. 1(b,c) respectively. These figures indicate that for both types of thin-pSAFs, it is possible to reduce the dipolar field for sub-20 nm memory cell to acceptable values below 200 Oe. However, to achieve less than 200 Oe of dipolar field on the storage layer, the required number of Co/Pt bilayers in the hard layer is 3 to 4 for the previous art thin-SAF and 2 to 3 for the novel thin SAF with multi-functional RKKY coupling layer. Therefore, the MF-AFC layers allow further reduction of the total thickness of the pSAF stack, which further eases the etching of this layer.

### RKKY coupling energy versus Ru/W thicknesses

**Figure 2:**
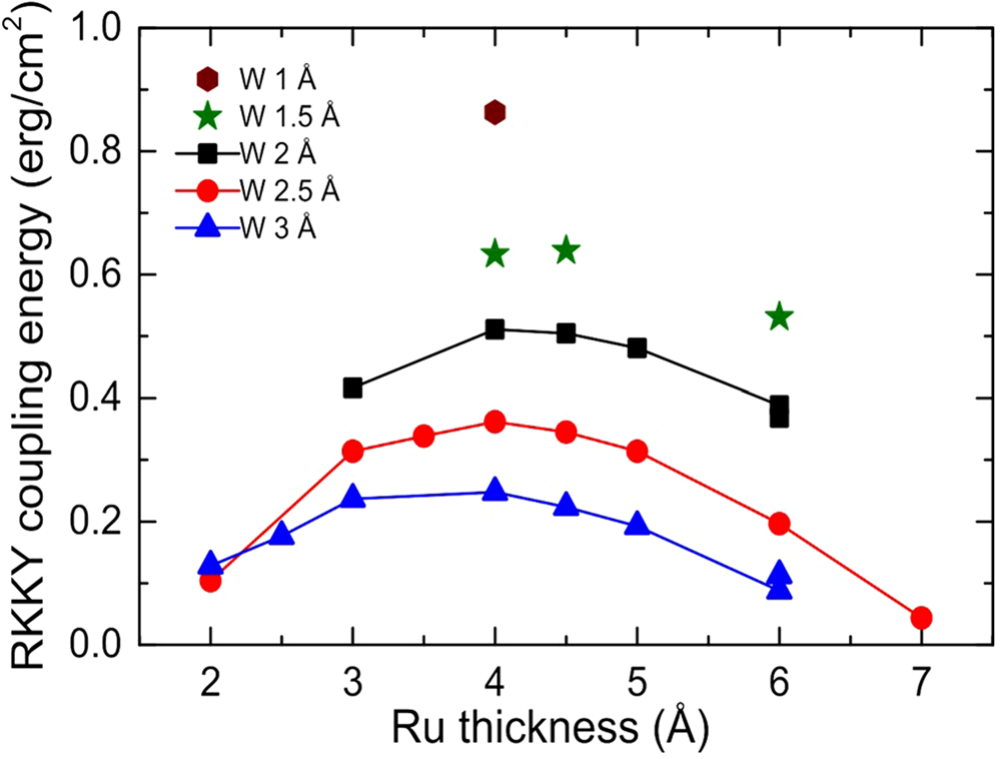
RKKY coupling energy density (J_RKKY_) of the pSAF versus thicknesses of Ru and W layers of MF-AFC after annealing at 340 °C.

**Figure 3:**
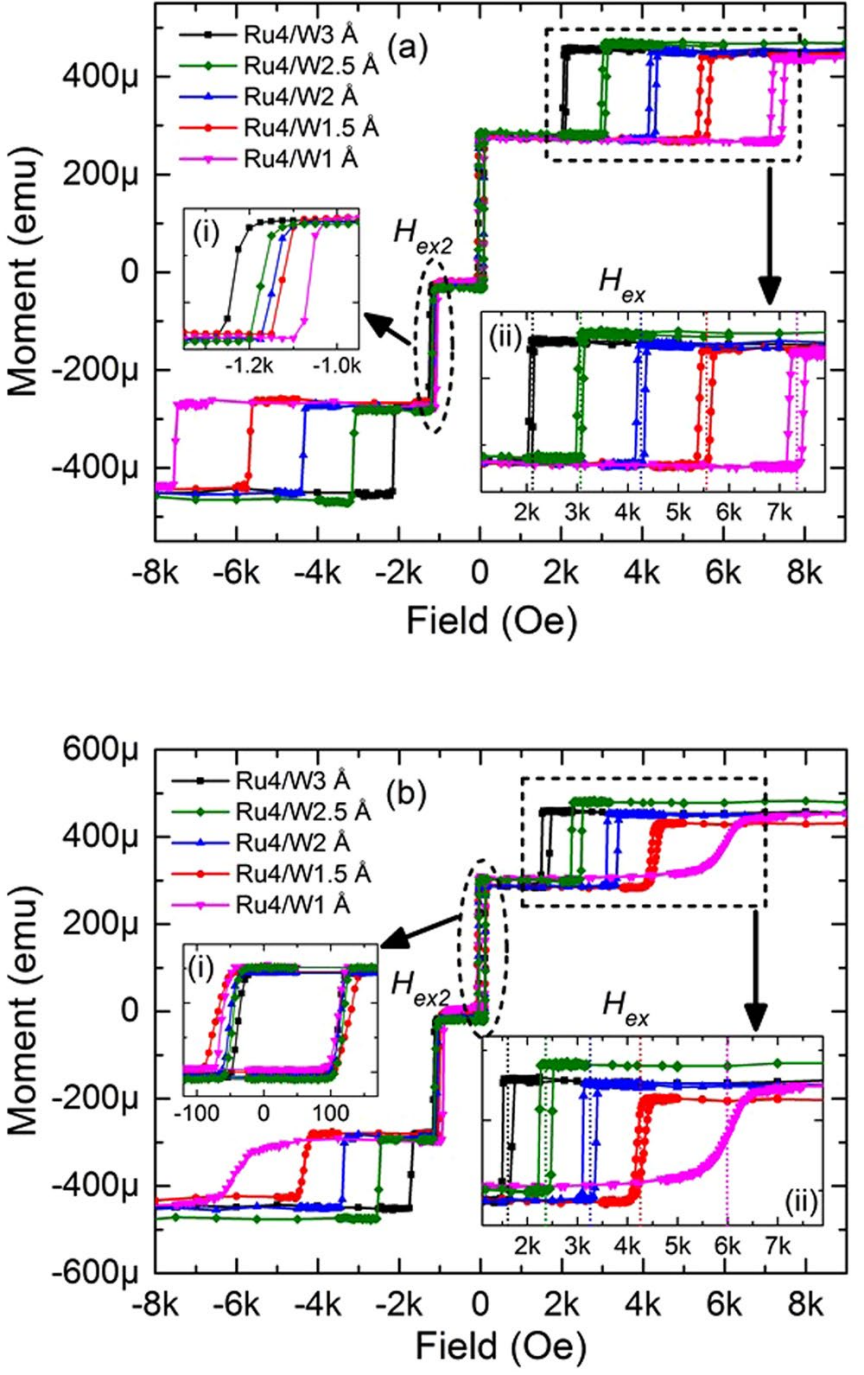
Descending branch of M(H) loops together with the minor loops of storage and polarizer layers of various pMTJ stacks (Si/Ta 3/Pt 5/[Co 0.5/Pt 0.25]_3_/Co 0.5/Ru 0.4/W (t_y_^)^/FeCoB 1.0/MgO/FeCoB 1.2/W 0.2/FeCoB 0.8/MgO cap/cap layers (thicknesses are in nm)) with different W thicknesses in MF-AFC, after annealing at (**a**) 340 °C and (**b**) 400 °C respectively. Inset (a–i) shows that the simultaneous reversal fields (H_ex2_) increase in absolute value with increasing W thickness while (b–i) shows the minor loops of the composite storage layers of the stacks. Inset (ii) of (**a**,**b**) are the minor loops of the FeCoB PL, showing that the interlayer exchange coupling field (H_ex_) increases with decreasing W thickness.

Thin pSAF layer with the configuration of *Si/Ta* 3*/Pt 5/*[*Co 0.5/Pt 0*.2*5*]_3_/*Co 0.5*/*Ru* (*t*_*x*_)*/W* (*t*_*y*_)*/FeCoB1.0/MgO/cap layer* (thicknesses are in nm) were deposited with various thicknesses of Ru and W. Supplementary Fig. S1 shows the descending branch of M(H) loops of the pSAF layer with Ru (t_x_)/W (1.5, 2, 2.5 and 3 Å) MF-AFC after annealing at 340 °C. Interlayer exchange coupling energy density (*J*_*RKKY*_) for different thicknesses of Ru and W has been calculated by the equation below and plotted in Fig. 2.

1 $${J}_{RKKY}={H}_{ex}{M}_{s}t$$

where *M*_*s*_ and *t* are the saturation magnetization and thickness of the FeCoB polarizer layer (PL). *H*_*ex*_ denotes the inter-layer exchange coupling field or RKKY coupling field, below which the polarizer layer and the Co/Pt hard layer (HL) are in anti-parallel alignment. The value of *M*_*s*_ of FeCoB was considered to be 1200 emu/cc. In addition, the magnetic field at which the first reversal of the thin-pSAF occurs along the descending branch of M(H) loops, as shown in Fig. S1, was considered as representing *H*_*ex*_ to calculate *J*_*RKKY*_ for different Ru and W thicknesses. However, more accurately *H*_*ex*_ should be chosen as the center point of the minor loops of PL as indicated in the insets (ii) of Fig. 3. However, we note that the hysteresis on the PL minor loop is much smaller than the loop shift so that the error on the J_RKKY_ determination reported in Fig. 2 for different W thicknesses is of the order of 1.5%, which can be considered in the error bar range. This figure demonstrates that, depending on the thicknesses of Ru and W layers, the coupling energy can be tuned over a broad range. The maximum RKKY coupling energy obtained is 0.86 erg/cm^2^ after 340 °C annealing, which is about 20% larger than the value calculated at the second peak of Ru (0.8–0.9 nm) for as-deposited pSAF and about 50% lower than the value obtained at the first peak of Ru (0.45 nm) after annealing at 350 °C^16^. However, one must note that the coupling energy reported by Yakushiji *et al*.^16^ is obtained for Ru sandwiched between two Co/Pt multilayers whereas the coupling energy that we measure with Ru/W is between a (Co/Pt) multilayer and amorphous/partly bcc FeCoB. For all W thicknesses, the peak in coupling energy occurs for Ru thickness in the range of 4 to 4.5 Å, which is the thickness region for the first peak of the oscillatory curve of RKKY coupling energy^17^.

Note that the Ru and particularly the W layers that are being used here are quite thin (1 to 3 Å thick). One cannot expect such thin layers to be spatially homogeneous in the layer plane. The reported values of coupling are therefore spatially averaged values. The spatial fluctuations may lead to higher order coupling terms which are not discussed here.

### Magnetic properties of thin-pMTJs after 340 °C and 400 °C annealing

Figure 3(a,b) show the descending branch of M(H) loops of several pMTJ stacks along with the minor loops of the storage and reference layers. The pMTJ stacks composition is *Si/Ta* 3*/Pt 5/*[*Co 0.5/Pt 0*.2*5*]_3_/*Co 0.5*/*Ru 0.4/W* (*t*_*y*_)/*FeCoB 1.0*/*MgO*/*FeCoB 1.2*/*W 0.2*/*FeCoB 0.8/MgO cap/cap layers* (thicknesses are in nm, *t*_*y*_ was varied between 0.1 and 0.3 nm). The samples were annealed at 340 °C, 400 °C respectively after deposition. Coming from high positive fields, the magnetization of FeCoB (1 nm) polarizer layer (PL) switches first at field slightly lower than *H*_*ex*_. *H*_*ex*_ becomes larger with decreasing W thickness, as illustrated in inset (ii) of Fig. 3(a,b) by the dotted lines. However, totally removing the W layer decreases the PMA of the FeCoB PL as well as the tunnel magnetoresistance amplitude as there is no more texture breaking layer between the bcc (100) FeCoB PL and fcc (111) Co/Pt MLs. As a consequence, the switching of PL becomes canted, as demonstrated in Supplementary Fig. S2. This proves the fact that one of the functions of the W layer of MF-AFC is to serve as a texture-breaking layer. When the field direction is reversed, after applying a very small negative field (−50 Oe), the magnetization of the SL is oriented along the field. With the increase of negative field, a characteristic field (*H*_*ex*2_) is reached at which the magnetic moments of both PL and HL reverse simultaneously to tail-to-tail orientation from head-to-head orientation. This reversal will be called concurrent reversal in the following of the paper. By decreasing the W thickness, as the interlayer exchange coupling energy is increased, *H*_*ex*2_ shifts towards lower field, which is shown in the inset (i) of Fig. 3(a). Finally the magnetization of the PL switches at higher field to align along the applied magnetic field. After annealing at 400 °C, as shown in Fig. 3(b), *H*_*ex*_ is slightly reduced most likely due to intermixing of Ru and W. As a result, the RKKY coupling layer with 1 Å W does not effectively break the texture. Therefore, the PMA of the PL becomes weaker. Hence, in order to maintain the PMA of the PL, the thicknesses of Ru and W layer should be optimized. In this case Ru 4/W 2 Å is the optimum thickness of MF-AFC which ensures stable magnetic properties of the pSAF. Besides, the inset (i) of Fig. 3(b) indicates that the composite storage layer used in these stacks can withstand 400 °C annealing temperature yielding square minor loop with high coercivity (≈80 Oe). Hence, these thin-pMTJ stacks with Ru 4/W 2 Å MF-AFC are back-end-of-line compatible.

### Controlling the concurrent reversal of these thin-pSAF

At this stage, the only concern is the occurrence of concurrent reversal of HL and PL magnetization at moderate field (*H*_*ex*2_ = 1100 Oe). This may cause the reference layer’s magnetization reversal during the writing of memory cell by spin transfer torque, and induce an error while writing. Therefore, *H*_*ex*2_ should be pushed towards higher fields or the associated transition be totally prevented to occur. In macrospin approximation, this concurrent reversal field can be viewed as the coercive field of the net moment of pSAF formed by the antiferromagnetically coupled sandwich of PL and HL via the RKKY coupling layer. Defining *M*_*HL*_, *K*_*HL*_, *t*_*HL*_ and *M*_*PL*_, *K*_*PL*_, *t*_*PL*_ as the magnetizations, perpendicular magnetic anisotropy and thicknesses of HL and PL respectively, *H*_*ex*2_ can be expressed as

$${H}_{ex2}\approx |\frac{{K}_{HL}{t}_{HL}+{K}_{PL}{t}_{PL}}{{M}_{HL}{t}_{HL}-{M}_{PL}{t}_{PL}}|$$

When *H*_*ex*_ > *H*_*ex2*_, the reversal of pSAF occurs in three steps. It is evident from the above expression, that *H*_*ex2*_ can be increased by increasing the anisotropy energy and/or by balancing the magnetic moments of the PL and HL. By doing so, when the condition *H*_*ex*_ < *H*_*ex2*_ is satisfied, the concurrent reversal step does not occur anymore.

**Figure 4:**
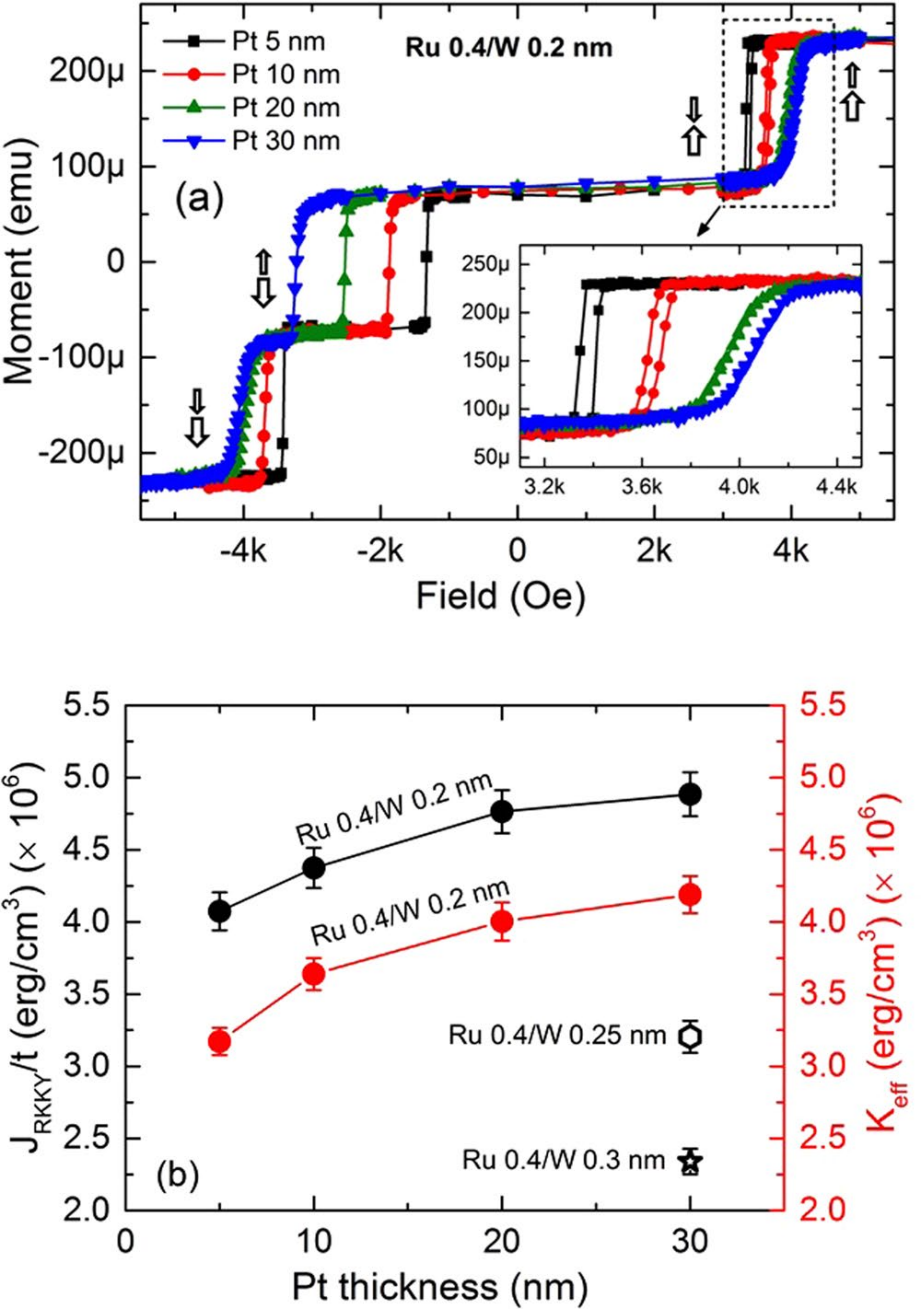
(**a**) Descending branch of M(H) loops of thin-pSAF of composition: Si/SiO_2_ 500/Ta 3/Pt (t)/[Co 0.5/Pt 0.25]_3_/Co 0.5/Ru 0.4/W 0.2/FeCoB 1.2/MgO/cap layers (thicknesses are in nm) with different Pt thickness, t = 5, 10, 20,30 nm after annealing at 340 °C. The arrows indicate the magnetization reversal process along the field-sweep from +6 kOe to −6 kOe. Inset of this figure displays the minor loops of FeCoB PLs. (**b**) Interlayer exchange coupling energy density (J_RKKY_/t) of thin-pSAFs and effective perpendicular magnetic anisotropy energy density (K_eff_) of HL as a function of Pt thickness. For the calculation of K_eff_, separate samples with the stack composition Si/SiO_2_ 500/Ta 3/Pt (t)/[Co 0.5/Pt 0.25]_3_/Co 0.5/Ru 0.4/W 0.2/Pt 3 nm were prepared. The error in K_eff_ has been calculated considering the error coming from magnetic moment and surface area measurements. The error in J_RKKY_/t has been calculated considering the error in H_ex_ as field step and error in M_s_ as described before.

**Figure 5:**
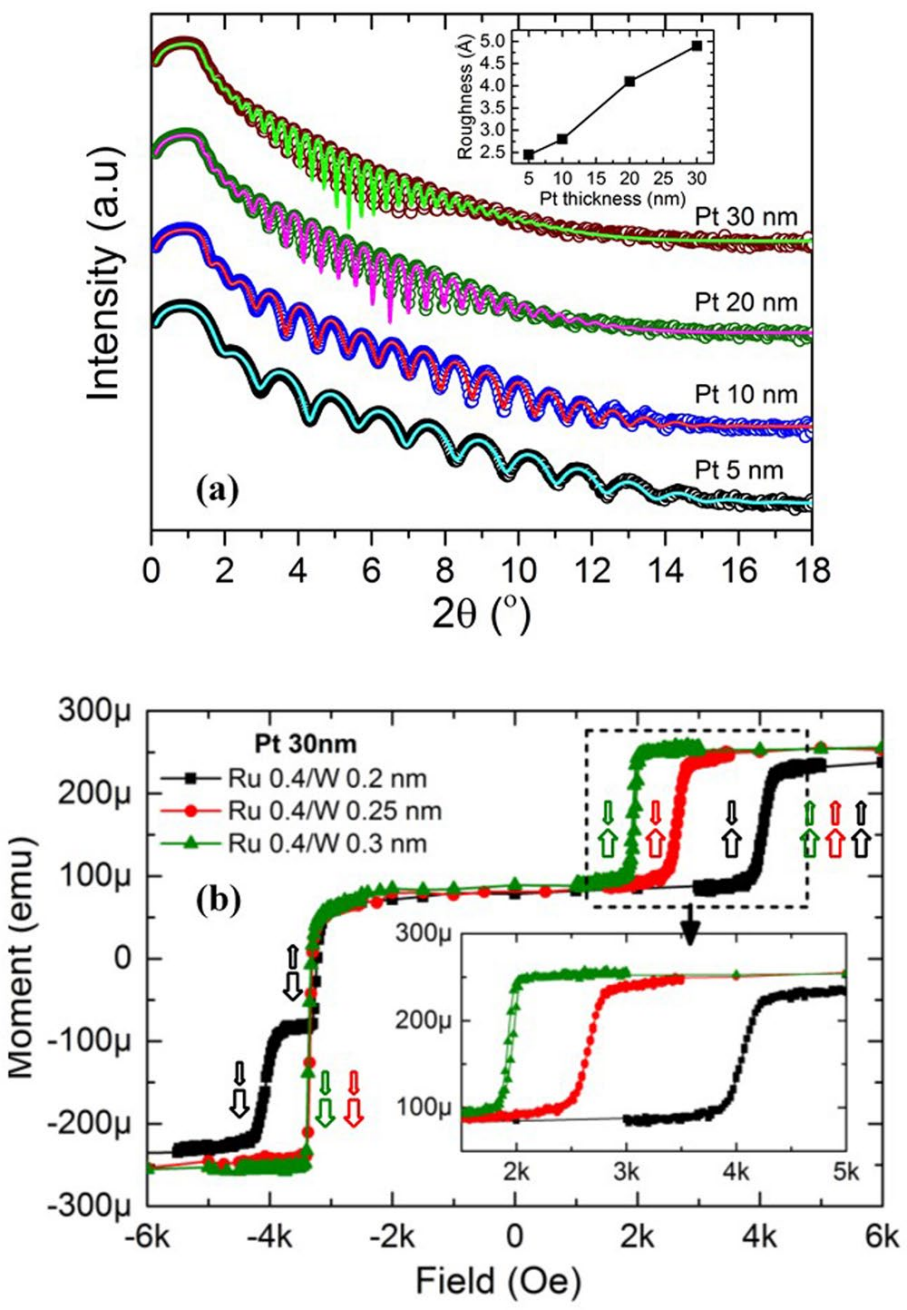
(**a**)X-ray reflectivity spectra of Pt seed layer with different thicknesses deposited on Si/SiO2 500 nm. Circular symbol and line plots represent the measured and simulated XRR spectra respectively. The inset describes surface roughness vs. Pt thickness, extracted from XRR fitting. (**b**) Descending branch of M(H) loops for three different thin-pSAFs when the magnetic field is swept out-of-plane from +6 kOe to −6 kOe. For the different samples, the reversal process is explained by arrows of corresponding colors. The layer composition of these stacks is (Si/SiO2 500/Ta 3/Pt 30/[Co 0.5/Pt 0.25]3/Co 0.5/Ru 0.4/W (t)/FeCoB 1.2/MgO/cap layers (thicknesses are in nm)) with different W thickness, t = 0.2, 0.25, 0.3 nm after annealing at 340 °C.

To illustrate the first option, i.e. to increase the *H*_*ex2*_ by improving the PMA of HL, four samples with the stack structure *Si/SiO2 500 nm/Ta 3/Pt* (*5,10,20,30*)*/*[*Co 0.5/Pt 0.25*]*3*/*Co 0.5/Ru 0.4/W 0.2/FeCoB 1.2/MgO*/*cap layer* (thicknesses are in nm) were deposited. The thickness of the Pt seed layer in these stacks was increased from 5 to 30 nm. Figure 4(a) shows the descending branch of hysteresis loops of the above mentioned four stacks after annealing at 340 °C. Concurrent reversal of PL and HL (*H*_*ex2*_) is present for all samples along with the spin flip (*H*_*ex*_). However, *H*_*ex2*_ increases with the thickness of Pt seed layer. Thicker seed layer improves the crystalline quality and the fcc (111) texture of the Co/Pt MLs. Therefore, the PMA of the HL increases^18^, which is presented in Fig. 4(b). With the increase of PMA, the hard layer becomes harder and the concurrent reversal (*H*_*ex*2_) appears at larger field. Despite the PMA improvement of HL, the concurrent reversal does not disappear as the energy density of interlayer exchange coupling (*J*_*RKKY*_*/t*) remains higher than the PMA energy density (*K*_*eff*_) even for the thickest Pt seed layer (30 nm), which is evident from Fig. 4(b). The interlayer exchange coupling is also improved with Pt seed layer thickness, most likely due to the improvement of crystallinity of Ru layer of MF-AFC. However, the reversal of FeCoB PL becomes slanted when the Pt thickness is 20 nm and larger. There are two reasons behind this behavior. First, the surface roughness of Pt seed layer increases with its thickness, which was extracted from the fitting of XRR spectra as described in Fig. 5(a). The inset of the figure shows that the roughness increases sharply when the thickness of Pt is larger than 10 nm. This surface roughness progresses throughout the Co/Pt MLs to the interface between FeCoB and MgO. Hence the PMA of the FeCoB PL is reduced. The second reason is the strong fcc (111) texture of [*Co 0.5/Pt 0.25*]_*3*_/*Co 0.5*/*Ru 0.4 nm* nanolaminates on thicker Pt seed layer, which 2 Å W layer cannot break efficiently. Therefore, the nano-crystallites of FeCoB layer do not crystallize strongly with bcc (001) texture. When the W layer of Ru/W MF-AFC layer is increased, the canting of the FeCoB reversal is reduced which is shown in Fig. 5(b). This indicates that the thickness of W layer must be optimized in order to maintain the texture breaking functionality of the MF-AFC. Moreover, the thickness of the Pt seed layer should be limited to a maximum thickness (10 nm) so that the surface roughness and the texture quality of the Co/Pt MLs do not become detrimental for the magnetic properties of the stack. For W thickness of 2.5 and 3 Å, the interlayer exchange coupling energy density (*J*_*RKKY*_/*t*) becomes less than the *K*_*eff*_ of the HL, which is shown in Fig. 4(b). In this case *H*_*ex*_ < *H*_*ex2*_ is satisfied. Hence, the simultaneous reversal process disappears, leading to two-steps magnetization reversals from positive to negative saturation. Coming from high positive field, the magnetization of FeCoB PL layer flips first and becomes anti-parallel with the HL. Due to different interlayer exchange couplings associated with Ru 0.4/W 0.25 and Ru 0.4/W 0.3 MF-AFC, this reversal occurs at 2670 Oe and 1970 Oe respectively. Then the hard layer magnetization reverses at −3350 Oe and saturates along the field direction.

**Figure 6:**
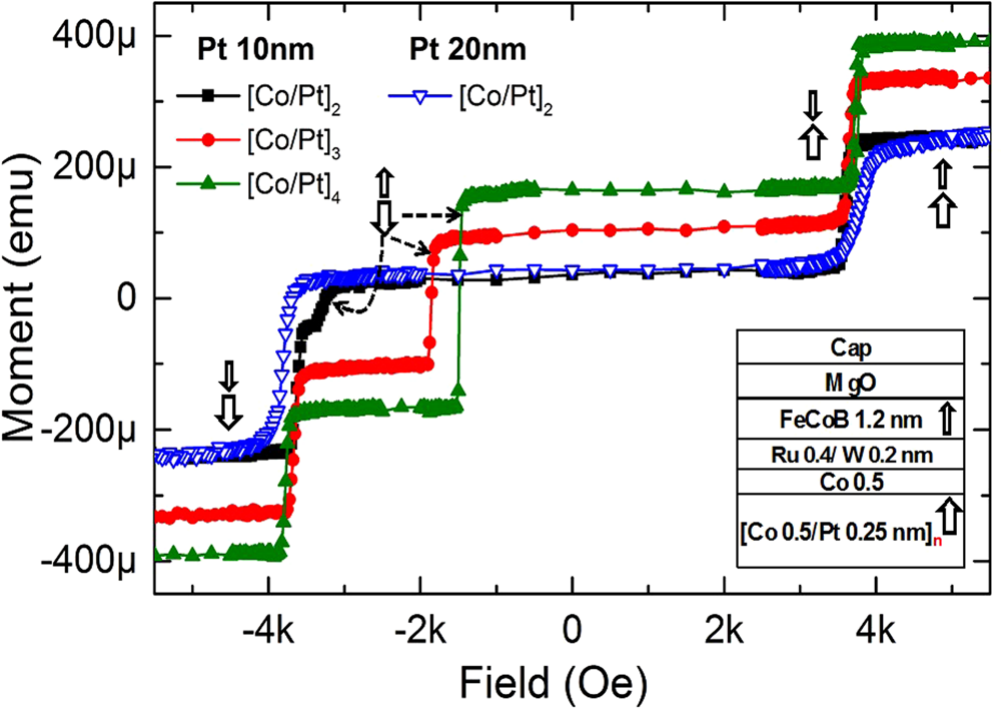
Descending branch of M(H) loops of thin-pSAFs with configuration of Si/SiO2 500/Ta 3/Pt 10 or 20/[Co 0.5/Pt 0.25]n_HL_/Co 0.5/Ru 0.4/W 0.2/FeCoB 1.2/MgO/cap layers (thicknesses are in nm) with different number of Co/Pt bilayers,(n_HL_ = 2, 3 and 4) after annealing at 340 °C. The arrows indicate the magnetization reversal process of the pSAF layers when the magnetic field is swept out-of-plane from +6 kOe to −6 kOe.

The second strategy consisting in balancing the magnetic moments of HL and PL was studied by changing the number of (Co/Pt) bilayers (n_HL_) from 4 to 2 in the HL. For this purpose, three samples of thin-pSAFs, (*Si/SiO2 500/Ta 3/Pt 10*/[*Co 0.5/Pt 0.25*]_***2,3 or 4***_/*Co 0.5/Ru 0.4/W 0.2/FeCoB 1.2/MgO*/*cap layer*, *thicknesses are in nm*) were characterized. Reducing the number of bilayers in the HL balances the magnetic moments of PL and HL. As a consequence, the denominator of the expression of *H*_*ex2*_ is reduced resulting in an increase in *H*_*ex2*_. The magnetic moment ratios between the HL and the PL (m_HL_/m_PL_) are 2.55, 1.96 and 1.42 respectively for n_HL_ = 4, 3 and 2. Correspondingly, concurrent reversal fields of 1475 Oe, 1850 Oe and 3300 Oe were measured, as reported in Fig. 6. When Pt 20 nm seed layer is used in combination with n_HL_ = 2, the condition *H*_*ex*_ < *H*_*ex2*_ is satisfied due to increase in PMA of HL. As a result, the concurrent reversal does not occur anymore. Another important point of balancing the magnetic moment is to reduce the dipolar field at the storage layer as illustrated in Fig. 1(f). With n_HL_ = 2 and 3, the dipolar field at the storage layer is expected to be lower than 150 Oe for sub-20nm memory cell which is shown in Fig. 1(f). Therefore, it is possible to realize an extremely thin, about 3.8 nm, magnetically stable pSAF layer ([*Co 0.5/Pt 0.25*]_*2*_/*Co 0.5*/*Ru 0.4/W 0.2*/*FeCoB 1.2 nm*) using the Ru/W MF-AFC.

### Electrical properties of thin-pMTJs

**Figure 7:**
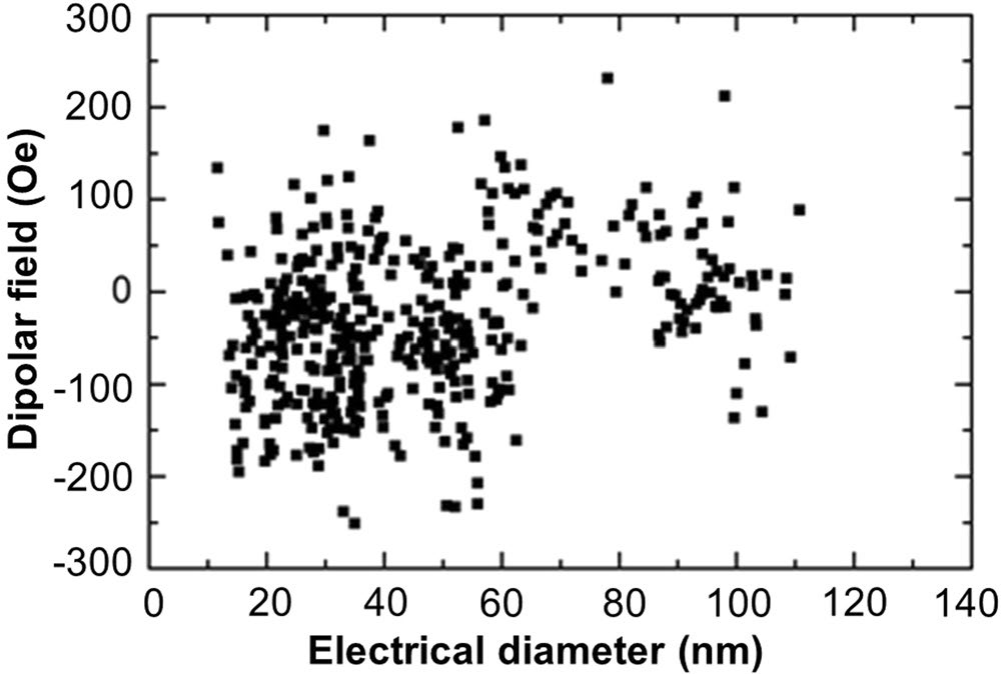
Dipolar field as a function of diameter of the memory cells. Remarkably, the stray field from the thin SAF on the storage layer remains below ~200 Oe for all cells which is quite low for pSTT-MRAM cells.

After having studied the magnetic properties at wafer level, a thin-pMTJ stack of composition *Si*/*SiO2 500*/*Bottom electrode*/[*Co 0.5/Pt 0.25*]_*3*_/*Co 0.5*/*Ru 0.4*/*W 0.2*/*FeCoB 1.2*/*MgO*/*FeCoB 1.5*/*W 2*/*top electrode* (thicknesses are in nm) was patterned to study the electrical properties of memory cells. The RA product of MgO barrier was 7.5 Ω.μm^2^. Before fabrication, the stack was annealed at 340 °C for 10 mins. The main purpose was to investigate the dipolar field at the storage layer and the stability of the FeCoB polarizer layer against applied write voltage pulses. Figure 7 shows the dipolar field at the storage layer as a function of electrical diameter of memory cells. This field is scattered due to process variability but exhibits values all lower than 200 Oe for sub-20 nm diameter memory cells, which is in agreement with the previous macrospin calculations.

**Figure 8:**
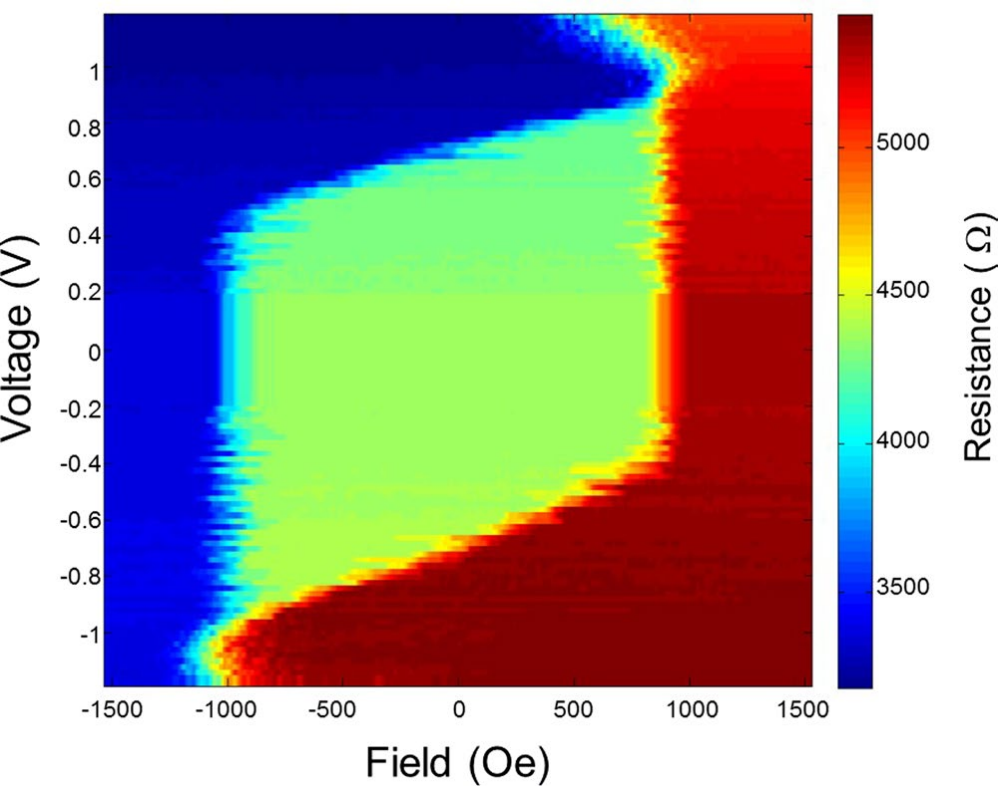
Voltage field phase diagram of a patterned cell with 53 nm electrical diameter. This diagram exhibits enough stability of the thin-pSAF consisting of FeCoB reference layer coupled with the [Co/Pt]x3 MLs against applied voltage pulse.

The stability of the reference layer of memory cells against applied write voltage pulse was characterized from voltage-field phase diagrams, one of which is displayed in Fig. 8. The device used for the phase diagram had an electrical diameter of 53 nm and 60% TMR. The width of applied voltage pulse was 100 ns. Figure 8 shows three resistance states. The region in red color corresponds to the high-resistance, where only the antiparallel (AP) configuration is stable. The low-resistance region in blue color represents the zone of stable parallel (P) state. The green zone in the middle corresponds to the bistable P/AP region, where the system can be either in AP or P states. The phase boundaries characterized by an almost linear variation of switching voltage versus field represent the influence of STT on the storage layer switching from AP to P or P to AP configuration. The other two vertical phase boundaries correspond to the storage layer coercive field. The parallelogram shape of this central part of the diagram indicates that the FeCoB reference layer’s magnetization remains unaffected by the current flowing through the stack (neither by Joule heating, nor by STT) up to 1 V, which is well above the critical switching voltage of the storage layer^19,20^.

## Conclusions

In conclusion, we have demonstrated a novel hybrid type multi-functional RKKY coupling layer combining Ru and W for STT-MRAM application. Apart from RKKY coupling the other functionalities exhibited by Ru/W MF-AFC are boron absorption from the FeCoB PL, texture breaking between HL and PL and reduction of interdiffusion of Ru into FeCoB at high annealing temperature. The maximum RKKY coupling energy obtained using Ru/W is 0.86 erg/cm^2^ after 340 °C annealing which is slightly larger than values obtained at the second peak of Ru (0.8–0.9 nm). The optimized thickness of Ru and W are 4 Å and 2 Å respectively to obtain BEOL compatible thin-pSAF. We have explained the importance of increasing the PMA of the HL and/or balancing the magnetic moments of HL and PL for improving the magnetic properties of the thin-pSAF layer. These optimizations enable to obtain an extremely thin, 3.8 nm thick pSAF layer ([*Co 0.5/Pt 0.25*]_*2*_/*Co 0.5*/*Ru 0.4/W 0.2*/*FeCoB 1.2* *nm*) with the interlayer exchange coupling field (*H*_*ex*_) of 3725 Oe and *H*_*ex*2_ of 3300 Oe. Functional STT-MRAM cells were patterned, demonstrating the stability of the reference layer against applied write voltage pulses up to 1 V and exhibiting very low dipolar field on the storage layer (<200 Oe) for sub-20nm electrical diameters. This novel class of MF-AFC is promising for STT-MRAM, SOT-MRAM and logic application.

## Methods

The samples were deposited by magnetron sputtering in a VAS sputtering tool with base pressure ~10^−8^ mbar under an Ar pressure during deposition of 2 × 10^*−*3^ mbar. The MgO tunnel barrier was obtained by naturally oxidizing a 0.7 nm thick metallic Mg layer under an oxygen pressure of 3 × 10^*−*2^ mbar with a flow rate of 100 sccm for 30 s. On top of this oxidized layer, a second Mg layer 0.5 nm thick was deposited. The resistance-area (RA) product for these oxidation conditions is 7 Ω *µ*m^2^. The composition of the magnetic electrode across MgO is Fe_72_Co_8_B_20_. All the samples were annealed for 10 min at different temperatures under a high vacuum (5 × 10^*−*6^ mbar). The magnetic properties were measured by using a vibrating sample magnetometer (VSM). The relatively high residual pressure of our deposition tool explains the moderate TMR amplitude of the samples used in this study (60%).

## Electronic supplementary material

Supplementary information
