# Supplementary material for: Novel multifunctional RKKY coupling layer for ultrathin perpendicular synthetic antiferromagnet
Source: Sci Rep. 2018 Aug 6;8:11724. doi: 10.1038/s41598-018-29913-6 (PMC6079069; doi:10.1038/s41598-018-29913-6)
Supplement: Supplementary file 1 — Supplementary information [file 41598_2018_29913_MOESM1_ESM.pdf]

Novel multifunctional RKKY coupling layer for ultrathin perpendicular synthetic antiferromagnet

Jyotirmoy Chatterjee, Stephane Auffret, Ricardo Sousa, Paulo Coelho, Ioan-Lucian Prejbeanu and Bernard Dieny

Univ. Grenoble Alpes, CEA, CNRS, Grenoble-INP, INAC-SPINTEC, Grenoble France

Magnetic cycles of thin-SAFs for different thicknesses of Ru/W

Thin-pSAFs with the layer configuration of *Si/Ta 3/Pt 5/[Co 0.5/Pt 0.25]3 /Co 0.5 /Ru* (t_x)*/W* (t_y)*/ FeCoB 1.0/MgO/cap layer* (thicknesses are in nm) were deposited with various thicknesses of Ru (t_x) and W (t_y). In these stacks *[Co 0.5/Pt 0.25]3 /Co 0.5 nm is the hard layer (HL) and FeCoB 1.0 nm is the polarizer layer (PL).* Figure S1 shows descending loop of out-of-plane M(H) loops of thin pSAFs with Ru(t_x)/W3, Ru(t_x)/W2.5 Ru(t_x)/W2 and Ru(t_x)/W1.5 Å RKKY coupling layers after annealing at 340°C. The M(H) loops were measured by vibrating sample magnetometer (VSM).

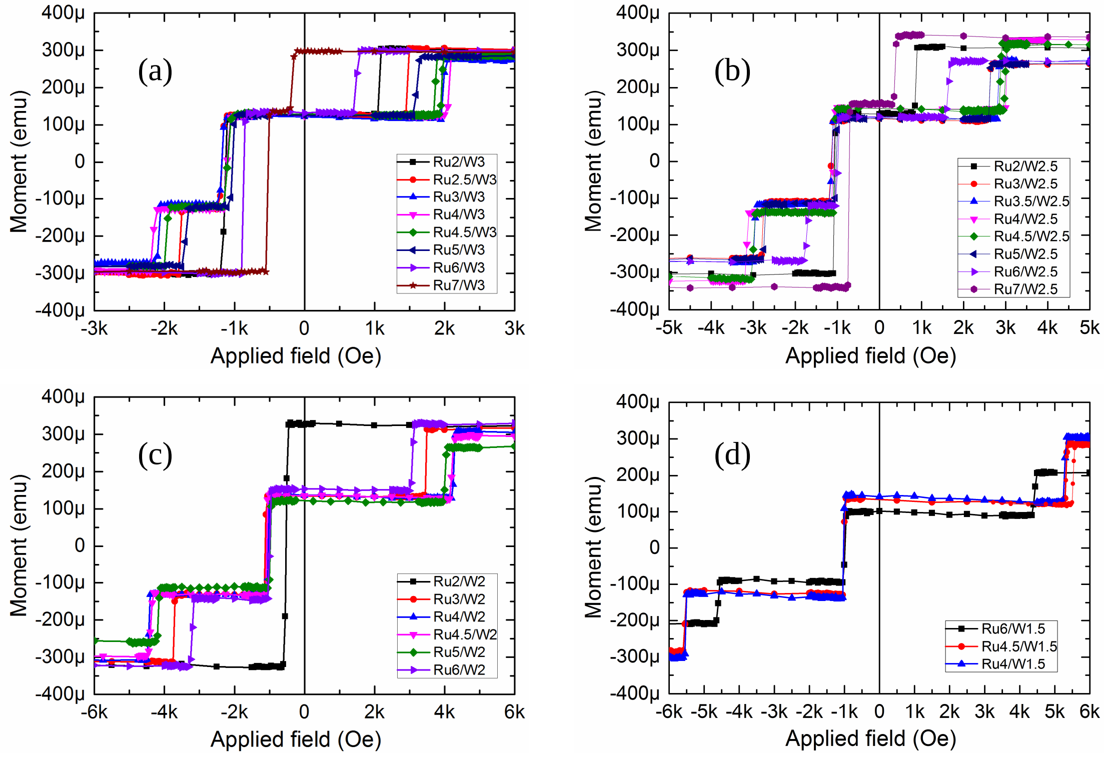

Fig. S1: Descending branch of out-of-plane M(H) loops of thin-pSAFs with stack structure of Si/Ta 30/Pt 50/[Co 5/Pt 2.5]3 /Co 5 /Ru (t_x)*/W (3, 2.5, 2 or 1.5)/ FeCoB 10/MgO/cap layer (thicknesses are in Å) after annealing at 340°C. (a) Ru* (t_x)*/W3, (b) Ru* (t_x)*/W2.5 (c) Ru* (t_x)*/W2 and (d) Ru* (t_x)*/W1.5 Å.*

All the magnetization reversal steps with the sweeping of magnetic field are sharp with high squareness signifying that the SAF layer possesses a well-defined perpendicular anisotropy.

Magnetic cycles of pMTJ stack with Ru 0.45 nm RKKY coupling layer

Fig. S2 demonstrate descending branch of M(H) loop of thin-pMTJ stack, the SAF layer of which antiferromagnetically coupled by only Ru 0.45 nm RKKY coupling layer. This stack consists of *Si/Ta 3/Pt 5/[Co 0.5/Pt 0.25]3 /Co 0.5 /Ru 0.4/FeCoB 1.0/ MgO/ FeCoB 1.2/ W 0.2/ FeCoB 0.8/MgO cap/top electrode* was annealed at 340°C after deposition. This figure presents canted reversal of FeCoB polarizer layer suggesting lack of perpendicular magnetic anisotropy in FeCoB layer due to the absence of a layer, which facilitates the transition from fcc (111) to bcc (001) crystalline planes.

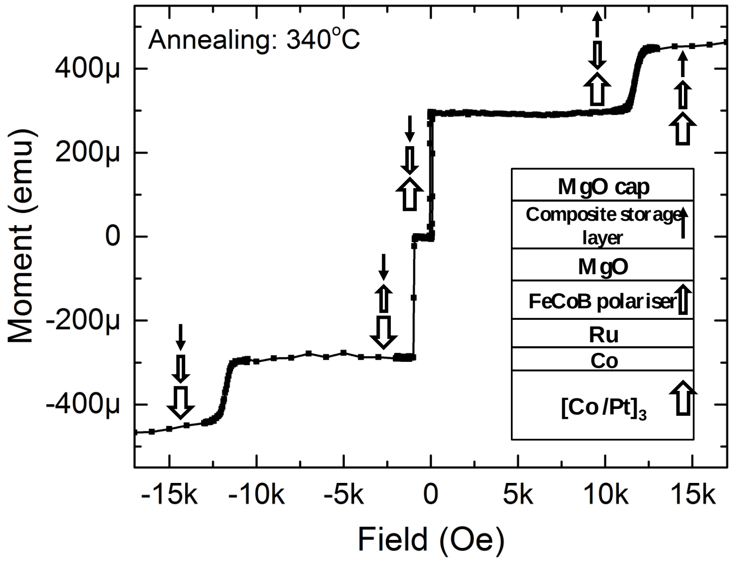

Figure S2. Descending branch of out-of-plane M(H) loops of thin-pMTJ stack (Si/Ta 3/Pt 5/[Co 0.5/Pt 0.25]3 /Co 0.5 /Ru 0.45/ FeCoB 1.0/ MgO/ FeCoB 1.2/ W 0.2/ FeCoB 0.8/MgO cap/cap layers (thicknesses are in nm)) after annealing at 340°C. The

arrows indicate the reversals of different magnetic components of the stack when the field is swept from 16 kOe to -16 kOe.
